# Nanoscale probing of electron-regulated structural transitions in silk proteins by near-field IR imaging and nano-spectroscopy

**DOI:** 10.1038/ncomms13079

**Published:** 2016-10-07

**Authors:** Nan Qin, Shaoqing Zhang, Jianjuan Jiang, Stephanie Gilbert Corder, Zhigang Qian, Zhitao Zhou, Woonsoo Lee, Keyin Liu, Xiaohan Wang, Xinxin Li, Zhifeng Shi, Ying Mao, Hans A. Bechtel, Michael C. Martin, Xiaoxia Xia, Benedetto Marelli, David L. Kaplan, Fiorenzo G. Omenetto, Mengkun Liu, Tiger H. Tao

**Affiliations:** 1 State Key Laboratory of Transducer Technology, Shanghai Institute of Microsystem and Information Technology, Chinese Academy of Sciences, Shanghai 200050, China; 2 Department of Mechanical Engineering, the University of Texas at Austin, Austin, Texas 78712, USA; 3 Department of Physics and Astronomy, Stony Brook University, Stony Brook, New York 11794, USA; 4 State Key Laboratory of Microbial Metabolism, School of Life Sciences and Biotechnology, Shanghai Jiao Tong University, Shanghai 200240, China; 5 School of Physical Science and Technology, ShanghaiTech University, Shanghai 200031, China; 6 Department of Neurosurgery, Huashan Hospital of Fudan University, Wulumuqi Zhong Road 12, Shanghai, 200040, China; 7 Nano-FTIR, Advanced Light Source Division, Lawrence Berkeley National Laboratory, Berkeley, California 94720, USA; 8 Department of Civil and Environmental Engineering, Massachusetts Institute of Technology, Cambridge, Massachusetts 02139, USA; 9 Department of Biomedical Engineering, Tufts University, Medford, Massachusetts 02155, USA; 10 Department of Chemical Engineering, Tufts University, Medford, Massachusetts 02155, USA; 11 Department of Physics, Tufts University, Medford, Massachusetts 02155, USA

## Abstract

Silk protein fibres produced by silkworms and spiders are renowned for their unparalleled mechanical strength and extensibility arising from their high-β-sheet crystal contents as natural materials. Investigation of β-sheet-oriented conformational transitions in silk proteins at the nanoscale remains a challenge using conventional imaging techniques given their limitations in chemical sensitivity or limited spatial resolution. Here, we report on electron-regulated nanoscale polymorphic transitions in silk proteins revealed by near-field infrared imaging and nano-spectroscopy at resolutions approaching the molecular level. The ability to locally probe nanoscale protein structural transitions combined with nanometre-precision electron-beam lithography offers us the capability to finely control the structure of silk proteins in two and three dimensions. Our work paves the way for unlocking essential nanoscopic protein structures and critical conditions for electron-induced conformational transitions, offering new rules to design protein-based nanoarchitectures.

## Abstract (web summary)

Silk protein fibres are exceptionally strong, owing to their high β-sheet nanocrystal content. Here, the authors use an electron beam to guide silk β-sheet crystals through structural transitions, and visualize the changes by infrared near-field optics, achieving close to molecular-level resolution.

## Graphical abstract

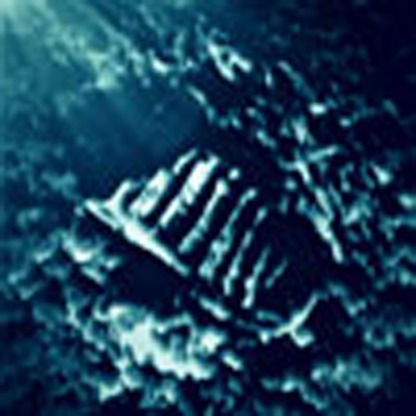

Proteins, the elementary building blocks of biological materials, possess many unique properties that are of fundamental importance to modern technology. Recent developments in nanotechnology have led to renewed interest and breakthroughs using biopolymers, specifically natural proteins, as novel functional materials^1^,^2^,^3^. In this context, silk has been heavily investigated because of its superior native mechanical properties (strength and toughness)^4^. In addition, silk-based biomaterials have a number of intriguing properties, such as outstanding biocompatibility and biodegradability^5^, controllable water-solubility^6^ and degradation rate^7^. These compelling traits arise from the hierarchical structures of well-organized β-sheet nanocrystals arranged in a semi-amorphous protein matrix^8^, enabling many important silk-based biomedical applications including, but not limited to, drug release^9^, degradable implants^10^, tissue engineering^11^ and regenerative medicine^5^. Therefore, understanding the mechanisms that underpin β-sheet formation and deformation as well as formulation of strategies to control inter- and intramolecular bonds within silk protein matrices is paramount for the control of protein structures and the improvement of material properties^12^,^13^. For example, silk was used either as a positive or negative electron-beam lithography (EBL) resist through interactions with electron beams given its polymorphic crystalline structure^14^. Different processing/preparation of silk proteins was required for use in positive or negative tone (for example, crystalline silk as positive resist and amorphous silk as negative resist). The inelastic collision of electrons with crystalline silk results in the formation of short polypeptides, which are water-soluble. While in negative EBL, using silk proteins where water radiolysis dominates, high electron beam doses are usually needed to form the intermolecular crosslinks to make the proteins water-insoluble.

Understanding the hierarchical formation of protein structures at their fundamental length scales will help to recognize essential nanoscopic protein structures and critical conditions for conformational transitions, which in turn provides insight into refined protein nanostructuring. However, conventional high-performance imaging techniques to characterize and recapitulate silk structure–function relationships at the nanoscale present challenges given their limitations in chemical sensitivity (for example, electron microscopy and atomic force microscopy (AFM)) or limited spatial resolution (for example, ‘far-field' infrared (IR) spectroscopy). Characterization of conformational changes in proteins can be carried out using IR scanning near-field optical microscopy (IR-SNOM). SNOM has been previously applied in the identification of spectroscopic signatures in a variety of solid state and polymer samples, including direct imaging of plasmon propagation on graphene^15^, nanoscale-mapping of phase transitions in correlated electron materials^16^, chemical identification of mineral polymorphs^17^ and secondary structure analysis of single-protein complexes^18^. Two types of SNOMs are widely used for protein study at the nanoscale: one for near-field imaging (that is, scattering-type SNOM, referred to hereafter as s-SNOM) and another for nano-spectroscopic studies (that is, thermal-expansion-based SNOM, referred to as AFM–IR), respectively. Built on an AFM, s-SNOM provides direct imaging and chemical identification of proteins with spatial resolution of ∼10 nm, significantly enhancing the ability to probe local chemical composites^19^. In comparison, with AFM–IR nano-spectroscopy, IR absorption causes a rapid local thermal expansion that excites resonant oscillations of the AFM cantilever, yielding frequency-dependent IR absorption spectra. Each absorption peak corresponds to a specific molecular resonance of the protein, providing a unique chemical fingerprint at the nanoscale^20^.

Here, we show electron-induced nanoscale structural transitions in silk proteins revealed by near-field IR imaging and nano-spectroscopy at resolutions approaching the molecular level. This work builds on the ability to reshape silk with energetic electrons and on the application of advanced spectroscopic imaging for nanoscale structural analysis and mapping. We directly visualize a complete structural transition (that is the formation, deformation, reformation, decomposition and carbonization) of β-sheet nanocrystals in silk protein thin films, controlled by EBL, unveiling an exciting route for high-level protein-based two-dimensional (2D) and three-dimensional (3D) nanofabrication and engineering.

## Results

### Silk proteins as dual-tone bio-resist in EBL

**Figure 1:**
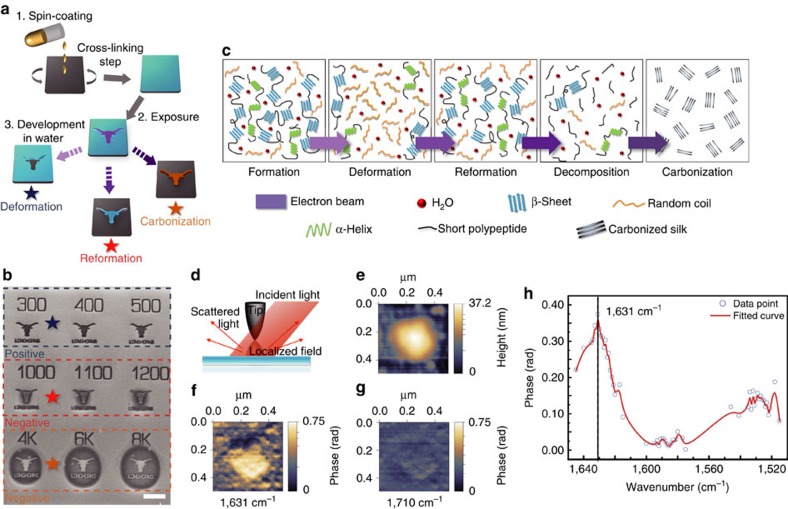
Electron-regulated nanoscale structural transitions in silk proteins. (**a**) Silk proteins as dual-tone bio-resist in EBL. (**b**) SEM images of nanopatterned crystalline silk as positive or negative resist on the same substrate (due to different structural transitions) depending on ebeam dosages. Scale bar, 5 μm. (**c**) Schematic illustration of β-sheet-oriented structural transitions regulated by electron energies. (**d**) Schematics of nanoscale IR spectroscopic imaging using scattering-type SNOM (s-SNOM). (**e**) Topography of silk nano-aggregates (β-sheet rich) on a silicon substrate. (**f**,**g**) Near-field IR phase images at 1,631 and 1,710 cm^−1^, respectively. (**h**) Local IR absorption spectra (symbols) depicting the normalized near-field phase signal of crystalline silk by sweeping the output wavenumber of the QCL and using IR nano-spectroscopic imaging.

We report here that either amorphous or crystalline silk (or intermediate conformational states) can be used in both positive and negative tones. The applied electron dosage is the primary tuning parameter and plays a more important role than the crystallinity of the starting materials (Supplementary Fig. 1). For example, positive and negative EBL were simultaneously achieved on the same crystalline silk protein substrate as the starting material undergoing an identical water-based resist development process (Fig. 1a,b). The control of polymorphic transitions in silk proteins allows us to explore a complete structural transition of β-sheet nanocrystals regulated by precise delivery of electron energies at the nanoscale (Fig. 1c).

### Nano-spectroscopic imaging of silk proteins using s-SNOM

In this work, SNOM has been utilized to overcome the diffraction limits of conventional optics and register nanoscale spectroscopic signatures of silk in the IR frequencies. To obtain high-resolution optical images and spectroscopic information to map out the nano-chemical and nano-mechanical properties of silk proteins at the molecular level, an s-SNOM (NeaSNOM, Neaspec GmbH, Germany) is coupled to a tunable IR quantum cascade laser (QCL, Daylight Solutions Inc., USA) covering the broad IR spectra of the amide I and II bands over the range from 1,495 to 1,790 cm^−1^ (Fig. 1d). The near-field phase spectrum resembles the molecular absorbance band, while the near-field amplitude spectrum acquires a dispersive line shape similar to a far-field reflectivity spectrum^21^. Figure 1e shows a topographic image of regenerated silk protein aggregates with high β-sheet contents, with sizes ranging from ∼10–350 nm, spin-coated on a silicon substrate (also see Supplementary Figs 2 and 3). Figure 1f,g show near-field IR phase images taken at 1,631 and 1,710 cm^−1^, respectively. All IR nano-imaging was performed at a spatial resolution of ∼10 nm approaching the molecular limit of silk proteins; for example, a *Bombyx mori* silk fibroin (∼7.8 nm)—as a model protein investigated in this work—consists of one light chain (∼2.4 nm) and one heavy chain (∼4.2 nm) linked by a disulfide bridge^22^. At 1,631 cm^−1^, the phase image exhibits a strong contrast between silk and silicon (silicon is used as the reference for IR imaging) owing to the amide I absorption corresponding to the secondary structure of β-sheets^23^. This phase contrast vanishes when the illumination is tuned to 1,710 cm^−1^ where silk proteins show little absorption. Local IR absorption spectrum (symbols) depicting the normalized near-field phase signal of crystalline silk (that is, β-sheet rich) was acquired using IR nano-imaging by sweeping the probing wavenumber/wavelength during nano-spectroscopic imaging (Fig. 1h). Non-invasive chemical and mechanical mapping of materials with nanometre scale resolutions is an ultimate goal in modern chemistry and material sciences: SNOM provides accurate nanoscale analysis of biomaterials (silk proteins in our case) at ambient conditions without the need of special sample preparation or causing significant structural changes—if any—during measurements (Supplementary Fig. 4).

### Nano-imaging of structural transitions in silk proteins

**Figure 2:**
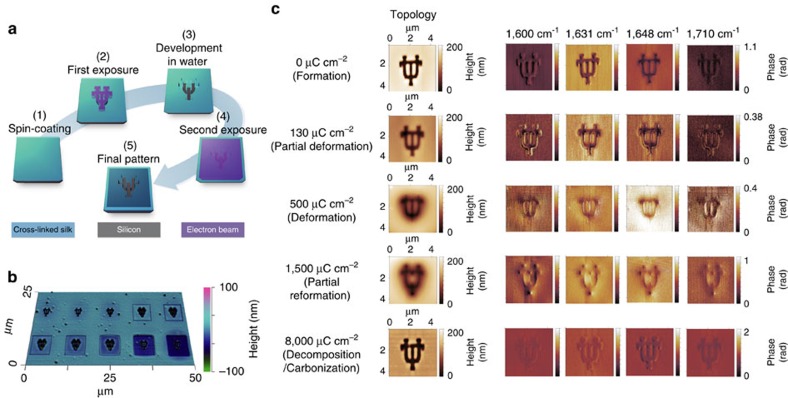
Direct visualization of electron-directed structural transitions of β-sheets using near-field IR nano-imaging. (**a**) Illustration of a two-step EBL process for sample preparation. First, an area of 3 × 3 μm (in the shape of a ‘UT' logo, line width: 200 nm) was patterned using EBL followed by a water development, which provided a clear contrast between silk and silicon and facilitated the following spectroscopic imaging/characterization. Then, a second step of electron irradiation was used to induce localized structural transitions in silk (5 × 5 μm squares) by delivering ebeams at various dosages. (**b**) AFM topographic images of silk nanopatterns fabricated using EBL at the dosages ranging from 0 to 8,000 μC cm^−2^. (**c**) IR nano-imaging using s-SNOM: the phase contrast between silk and silicon (a flat spectral response in the mid-IR) in each IR image correlates to the absorption of silk proteins (that is, the surrounding area of ‘UT' logos) of various structures at that wavenumber, and the comparison of contrast differences between the IR images (for instance, those in column 3 and column 4) implies the dominant protein structure within the amide I vibration bands (for example, 1,631 cm^−1^ for β-sheets and 1,648 cm^−1^ for random coils).

To explore the nanoscale conformational transition of silk proteins (with emphasis on the secondary structure of β-sheets), we prepared a set of silk fibroin samples on silicon substrates using EBL which offers the highest lithographic resolution at the nanoscale. A set of thin silk films with a thickness of ∼150 nm were spin-coated and crosslinked using methanol for crystallization (that is, the formation of β-sheets from random coils)^24^. A reference substrate (for example, silicon or gold) with a flat IR response is typically needed in s-SNOM measurements^25^. A two-step EBL was therefore applied (1) to create a silicon pattern of ‘University of Texas at Austin (UT)' in the first step EBL after a water development; and (2) to expose a silk pattern on a square-shaped, carved out ‘UT' region of the substrate, irradiated with different dosages of electrons in the second step EBL, for electron-induced conformational transition characterization (Fig. 2a). The ‘UT'-shaped silicon substrate served as an IR reference and facilitated both topographic characterization and more importantly IR nano-imaging. Multiple samples were prepared to elucidate the fundamental structural variations of silk samples by systematic exposures of silk samples to different doses of electron-beam radiation. Notable differences have been found in terms of the sharpness (Supplementary Fig. 5) and thickness (Fig. 2b) of as-fabricated silk nanostructures that underwent the radiolysis and pyrolysis processes—dominant at low and high dosages—respectively.

Figure 2c illustrates the IR nano-imaging of the formation (induced by chemical treatment using methanol^9^), deformation, reformation, decomposition, and carbonization (all induced by electron radiation) of β-sheet contents in silk nanostructures using s-SNOM. The first column shows the topographic images. Both sets of IR phase images—which were normalized to the silicon substrate—of silk nanostructures taken at 1,600 cm^−1^ (column 2) and 1,710 cm^−1^ (column 5) show weak contrast between silk and silicon, indicating an off-resonant response of the amide I bands. At the dosage of 0 μC cm^−2^ (no electron irradiance), the phase image taken shows a strong contrast at 1,631 cm^−1^ for β-sheets (column 3), which is much higher than the contrast in the image taken at 1,648 cm^−1^ for random coils (column 4, characteristic peak for amorphous silk, Supplementary Fig. 6), indicating a dominant β-sheet existence in crystalline silk. The difference in the phase contrast between images taken at 1,631 and 1,648 cm^−1^ slightly decreases at the dosage of 130 μC cm^−2^, indicating the partial deformation of the β-sheets (which transformed to unordered amorphous silk) in crystalline silk. When the dosage is increased to 500 μC cm^−2^, the image taken at 1,648 cm^−1^ shows a noticeably higher contrast than the one taken at 1,631 cm^−1^, opposite to the case of 0 μC cm^−2^ dosage, suggesting a typical organization of the unordered amorphous protein from a more complete deformation of β-sheets. With increasing electron beam dosage to 1,500 μC cm^−2^ the phase images taken at 1,631 cm^−1^ show a marginally higher (but comparable) contrast to 1,648 cm^−1^, which is believed to be due to a partial reformation of β-sheets from unordered silk polypeptides (that is, re-crystallizing). Partial recrystallization has been observed in previously reported work using chemical^26^,^27^ or thermal treatments^28^.

At the dosage of 8,000 μC cm^−2^, no substantial contrast was found in the phase images at the four frequencies. This is attributed to the decomposition of β-sheets along with a partial formation of carbonaceous pyroprotein after excessive electron irradiance treatment of β-sheet nanostructures, as indicated by the increased IR reflectivity of a more developed carbon structure at higher dosages (Supplementary Fig. 7). These results are similar to the previously reported macroscale carbonization of β-sheet-rich silk protein by heat^29^,^30^. This finding offers a potential method for direct formation of nanopatterned carbon structures using polymer based materials^31^ by controlling the protein thickness and electron beam dosage.

### Nano-spectroscopy of structural transitions in silk proteins

**Figure 3:**
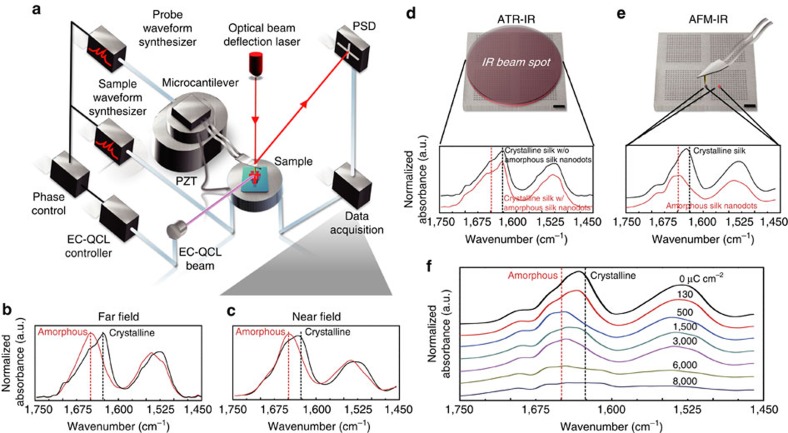
Quantitative evaluation of conformational transitions in silk proteins using near-field IR nano-spectroscopy. (**a**) Schematics of IR nano-spectroscopy using AFM–IR: pulses of IR radiation emitted by an IR QCL (output range: 1,460–1,780 cm^−1^, swept by a step size: 1 cm^−1^) were used to illuminate the sample, causing a rapid thermal expansion of silk nanostructures due to local absorption enhancement at various stages picked by the AFM tip, corresponding to the absorption spectroscopic signatures. (**b**,**c**) The AFM–IR spectra on amorphous and crystalline silk thin films are consistent with the conventional bulk FTIR spectra. (**d**,**e**) Spectra of a crystalline silk thin film with embedded amorphous silk nanopatterns of ∼30 nm fabricated using EBL, characterized by attenuated total reflection IR (ATR-IR) and AFM–IR, respectively. AFM–IR offers a considerable advancement ( × ∼1,000improvement spatially) in distinguishing nanoscale structural heterogeneity. (**f**) AFM–IR spectra of electron-induced structural transitions in silk proteins. PSD, position sensing detectors; PZT, lead zirconate titanate (Pb[Zr_x_Ti_1-x_]O3); EC- QCL, external cavity quantum cascade laser.

To quantitatively confirm the conformational transition and acquire unambiguous structural identification of each stage, we performed an IR nano-spectroscopy study of the electron-induced structural transitions of silk proteins using AFM–IR with a spatial resolution of ∼20 nm (Anasys Instruments, USA)^32^. IR pulses emitted by an IR QCL (Daylight Solutions Inc., USA; output range: 1,460–1,780 cm^−1^, swept by a step size of 1 cm^−1^) were used as the near-field source to illuminate the sample, causing a rapid thermal expansion of silk nanostructures corresponding to the absorption fingerprints (Fig. 3a). The AFM–IR spectra on amorphous and crystalline silk thin films are consistent with the conventional bulk Fourier transform infrared spectroscopy (FTIR) spectra (Fig. 3b,c). However, AFM–IR offers an important advancement (∼1,000 × improvement in the spatial resolution) as compared with previously reported work using conventional IR techniques^28^, which average the structural information over relatively large areas (that is, a few microns to a few dozen microns using FTIR-microscopy) on silk materials with high structural heterogeneity at the nanoscale (Fig. 3d,e). Crystalline silk shows a maximum absorption at ∼1,625 cm^−1^ (β-sheets) with two shoulder peaks at ∼1,645 cm^−1^ (random coils) and ∼1,660 cm^−1^ (α-helices), in good agreement with the frequency ranges corresponding to vibrational bands in β-sheet-rich *B. mori* silk within the amide I region of the spectrum^33^. Note that the resonance peak may differ within 10 cm^−1^ in s-SNOM and AFM–IR, as has been previously observed in s-SNOM spectra when compared to far-field IR and thermal-expansion-based AFM–IR spectroscopies as a result of tip-sample coupling^34^ and the spectral phase approximation^35^.

As shown in Fig. 3f, the characteristic peak intensity of the β-sheet formation at ∼1,625 cm^−1^ decreased as the dosage increases from 0 to 500 μC cm^−2^, indicating the continuing deformation of the β-sheet content and a slight increase of α-helix regions. After increasing the dosage to 1,500 μC cm^−2^, a resurgence of the absorption intensity correlated to the β-sheet formation at ∼ 1,625 cm^−1^ was present, which was noticeably lower than the original peak in crystalline silk, indicating partial reformation of β-sheets. The AFM–IR spectra of silk nanostructures under excessive electron dosage revealed that the characteristic peaks for β-sheet crystal structure were gradually weakened and broadened as the dosage increased from 1,500 to 6,000 μC cm^−2^ and disappeared following an electron irradiance at 8,000 μC cm^−2^, indicating that the β-sheet crystals were progressively decomposed and carbonized at high dosages (Supplementary Fig. 8). The changes in the characteristic peaks of the silk proteins indicates a more significant decrease in the fraction of amorphous regions relative to the β-sheet regions. A detailed deconvolution of the amide I band was conducted (Supplementary Fig. 9) and the secondary structure content of each stage was quantified (Table 1). In addition, we observed a noticeable difference in the structural integrity of silk proteins after electron irradiation. The crystalline and decomposed (partially carbonized) silks show considerably better pattern fidelity (namely higher sharpness, column one in Fig. 2c), which we hypothesize to be partially due to their highly ordered structures^29^ and applicable stray exposure (that is, proximity effects) caused by the backscattered electrons through the exposed silicon substrate (Supplementary Figs 10–14).

### Electron–structure interactions in silk proteins

**Figure 4:**
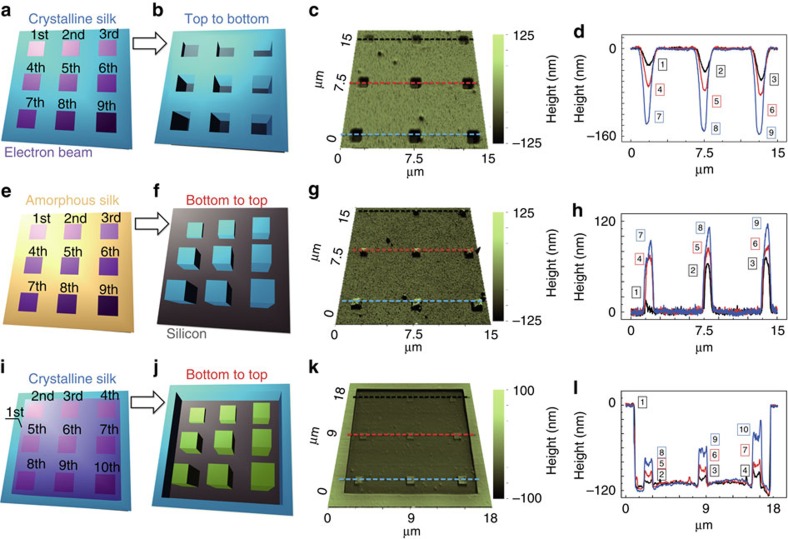
Electron-structure interactions in amorphous and crystalline silk proteins. (**a**–**l**) Three sets of silk nanostructures have been made in positive (row 1, nanosculpturing) and negative (row 2 and row 3, nanosintering) tones using EBL at various dosages. The lineouts (column 4) correspond to the dashed lines in AFM topographic images (column 3). A pre-exposure was applied to deform/de-crosslink β-sheets (to random coils) in crystalline silk (row 3), which can be further re-crosslinked to form negative nanostructures, similar to those generated by the process starting with the amorphous silk (row 2).

The ability to structurally characterize the material allows us to conduct a comprehensive evaluation of silk proteins for 3D nanostructuring. We found that there is a significant difference in the kinetics of protein–electron interactions between amorphous silk (random coil dominated) and crystalline silk (β-sheet dominated) (Fig. 4). In this report, we demonstrate fabrication of 3D silk nanostructures by *in situ* altering conformational structures of proteins using EBL with two different but complementary methods, namely electron-nanosculpturing (a subtractive manufacturing process, Fig. 4a–d) and electron-nanosintering (an additive manufacturing process, Fig. 4e–h). Note that crystalline silk can be also used in electron-nanosintering but an initial EBL exposure for β-sheet deformation is needed (Fig. 4i–l).

As revealed by near-field IR imaging and nano-spectroscopy, the interaction between the electron beam and the silk structure critically depends on the structural conformation on the protein matrix and as-applied electron dosage (Supplementary Fig. 15). For crystalline silk exposed to the electron beam, scission of the crosslinked β-sheets tends to occur from top to bottom, resulting in the removal of materials after a water-based development, which is referred to as electron-nanosculpturing. In contrast, for the amorphous silk exposed to the electron beam, crosslinking of unordered random coils (either intrinsic or deformed from crystalline proteins upon electron irradiations) proceeds from bottom to top, which is referred to as electron-nanosintering. The ability to understand basic mechanisms of electron-induced structural transformations allows us to produce sophisticated nanotopographies and nanostructures (Supplementary Figs 16–19), opening up numerous opportunities including biomimetic nanosurfaces^36^ and tissue engineering applications^37^.

### 2D and 3D nanostructuring of silk protein films using EBL

**Figure 5:**
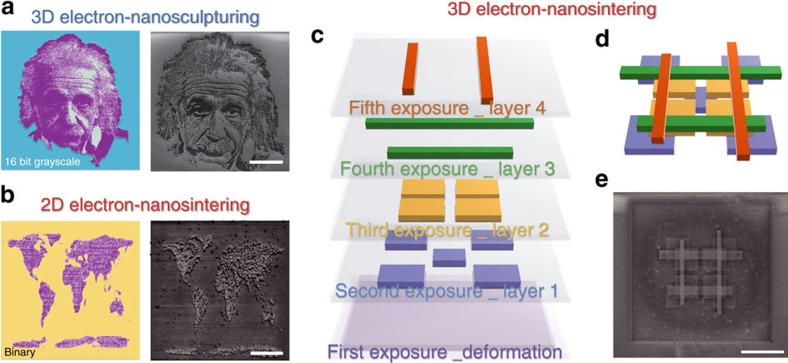
2D and 3D nanostructuring of silk proteins. (**a**) 3D electron-nanosculpturing: 3D nanotopographies on crystalline silk can be created using a 16-bit grayscale positive EBL (left: design image; right: SEM image). The Einstein image: Photo by Philippe Halsman @ Halsman Archive. One grayscale exposure was applied, followed by a water-only development to remove the exposed area. Scale bar, 10 μm. (**b**) 2D electron-nanosintering: 2D nanotopographies on amorphous silk proteins can be created using a binary negative EBL (left: design image; right: SEM image). Scale bar, 15 μm. One exposure was applied to crosslink the exposed area. Unexposed area is removed after water development. (**c**) 3D electron-nanosintering: 3D nanostructures on crystalline silk can be created using a LbL multi-EBL. Multiple exposures are applied in sequence to define each layer. The first exposure is to de-crosslink the crystalline silk proteins, resulting in amorphous proteins to be sintered/re-crosslinked by the following LbL EBL steps. For amorphous silk, this step (the first exposure) is unnecessary. (**d**) Schematic and (**e**) SEM images of as-designed 3D silk nanostructures using an LbL nanosintering process. Scale bar, 5 μm.

Several examples were fabricated as the first proof-of-principle demonstrations (Fig. 5). While the results express some resemblance to those by multi-photon polymerization (MPP) technique^38^, our methods differ in two important aspects. First, our fabrication is not limited by the optical diffraction (∼100 nm in advanced MPPs, estimated by Abbe's equation) but by the electron diffraction (<10 nm in standard EBLs, estimated by the de Broglie equation), offering significant improvements in achievable structuring resolutions. Second, photo initiators were required to enhance MPP in silk fibroin proteins^38^ while our techniques deal with pure silk in an all-water-based process, better preserving the biocompatibility of the material. Low throughput has been the fundamental limit of EBL despite its unparalleled lithographic resolution. For example, it took ∼5 and 10 min to fabricate the grayscale Einstein image (∼35 × 35 μm) and the layer-by-layer (LbL) multilayer structure (∼16 × 16 μm) shown in Fig. 5, respectively. Nevertheless, nanoprobing of electron-beam induced protein structural transitions using near-field spectroscopic imaging techniques reported in this work can be readily extended to study conformational dynamics of a variety of proteins (for example, keratins, collagens, and spider silk proteins^40^, data not shown) using other conventional nanofabrication systems/sources (for example, ion beams and photons, data not shown).

This comprehensive investigation of the electron-beam induced conformal modification of silk at the nanoscale using IR near-field optics, allows the characterization of the structural transitions of silk proteins upon electron irradiation. A deep understanding of the structure-property relation in protein-based biomaterials unveils an exciting route for high-level protein-based 3D nanofabrication and engineering, opening up possibilities for a new set of biomaterials with performance and function unattainable with other materials.

## Methods

### Preparation of silk fibroin proteins

Silk fibroin proteins were prepared using the established purification protocols^39^. *B. mori* cocoons were boiled for 30 min in aqueous 0.02 M Na_2_CO_3_ (Sigma-Aldrich, USA) and then rinsed for 3 × 30 min in distilled water to remove the Na_2_CO_3_ and sericin. The degummed cocoons were allowed to dry for more than 12 h and then subsequently dissolved in 9.3 M LiBr (Sigma-Aldrich, USA) solution at 60 °C for 4 h. The solution was dialysed for 2 days in distilled water using Slide-a-Lyzer dialysis cassettes (MWCO 3,500, Pierce, USA). The solution was centrifuged for 2 × 20 min at 18,000 r.p.m. The concentration was determined by measuring a volume of solution and the final dried weight.

### Sample preparation

The silk solution was spin-coated on silicon wafers. Thickness can be controlled by the spin speed and the concentration of the silk solution. In our case, 150-nm-thick silk layers were produced by spin-coating 5% silk fibroin solution at a maximum speed of 4,000 r.p.m. for 40 s. Crosslinking (that is, crystallization) of the film was obtained by dipping it in methanol for 5 min. An EBL tool (Hitachi-4800) was used to expose the silk protein thin films. For most samples shown in this work, the doses used varied from 0 to 8,000 μC cm^−2^ at 25 keV with a probe current of 10 pA.

### Nanoscale infrared spectroscopic imaging using s-SNOM

We utilized a commercially available scattering-type near-field microscope (s-SNOM, Neaspec GmbH, Germany) with a QCL IR laser (MIRCat, Daylight solutions Inc., USA) that is tunable between 1,495 and 1,790 cm^−1^. During instrument operation, the laser was attenuated to ∼10 mW such that the detector yields a nominal signal of 1.5 V. The AFM was operated in tapping mode with 65 nm tapping. Gold-coated AFM tips with about 250 kHz resonance (Tap300G-B-G, budgetsensors.com) were used to enhance the IR signal. The IR signal was detected simultaneously with AFM signals. The IR signal used for analysis in this work was measured by a lock-in amplifier at the second and third harmonics of the tapping frequency and the pseudo heterodyne technique, which provides both reflection and absorption that are (mostly) free of background. The image was scanned at 3.3 ms per pixel for a 500 × 500 pixel sized image.

### Infrared nano-spectroscopic study using AFM–IR

The IR spectrum was acquired using an AFM–IR system (Anasys Instruments, CA, USA). It allows high spatial and spectral resolution IR absorption measurement using a combination of AFM and IR laser source. The AFM measures the local thermal expansion of the sample due to the absorption of IR laser, and thereby maps material absorption as a function of wavenumber. Topography images were scanned before the IR spectra measurement to precisely locate the point of interest. The spectrum was acquired in the range between 1,460 and 1,780 cm^−1^, with a spectral resolution of 1 cm^−1^ using multi-region laser power settings to ensure consistent signal to noise ratio. The spectrum data was averaged by 10 repeated scans on the same spot. Also, it was averaged by taking five measurements on adjacent spots (each with 10 scan averaging) with the same composition. The sample data was normalized with respect to the spectrum of silicon under the same ambient environment (∼18% humidity and room temperature). Subsequently, a simple 10-point smoothing algorithm was performed to obtain the smooth near-field spectra.

### Decomposition of the amide I band

The band decomposition was performed with the OPUS software package (version 4.2) supplied by Bruker. As a starting point for the curve-fitting procedure, four individual absorption bands were proposed at 1,625, 1,645, 1,660 and 1,680 cm^−1^, defining β-sheets, unordered random coils, a-helices and β-turns structures, respectively. The curve fitting was successfully performed based on the damped least squares optimization algorithm (>99.9%) developed by Levenberg–Marquardt and assuming Gaussian band envelopes.

### Data availability

The data that support the findings of this study are available from the corresponding author (T.H.T., tiger.tao@austin.utexas.edu) upon request.

## Additional information

**How to cite this article:** Qin, N. *et al*. Nanoscale probing of electron-regulated structural transitions in silk proteins by near-field IR imaging and nano-spectroscopy. *Nat. Commun.* **7,** 13079 doi: 10.1038/ncomms13079 (2016).

## Supplementary Material

Supplementary Information Supplementary Figures 1-19.

## Figures and Tables

**Table 1:** Quantification of the nanoIR spectra using deconvolution (Percentage: %).

**Dosages (μC cm^−2^)**	**β-sheet**	**Random coil**	**α-helix**	**β-turn**	**Sum**
0	35.20	30.50	23.50	10.80	100.00
130	29.24	28.26	20.41	11.23	89.13
500	10.74	25.52	23.36	12.47	72.09
1,500	19.10	21.05	16.26	11.07	67.48
3,000	15.20	22.64	16.58	11.38	65.81
6,000	6.56	11.05	10.44	7.59	35.65
8,000	6.34	6.98	7.26	5.08	25.66
