# Supplementary material for: Nanoscale probing of electron-regulated structural transitions in silk proteins by near-field IR imaging and nano-spectroscopy
Source: Nat Commun. 2016 Oct 7;7:13079. doi: 10.1038/ncomms13079 (PMC5059764; doi:10.1038/ncomms13079)
Supplement: Supplementary Information — Supplementary Figures 1-19. [file ncomms13079-s1.pdf]

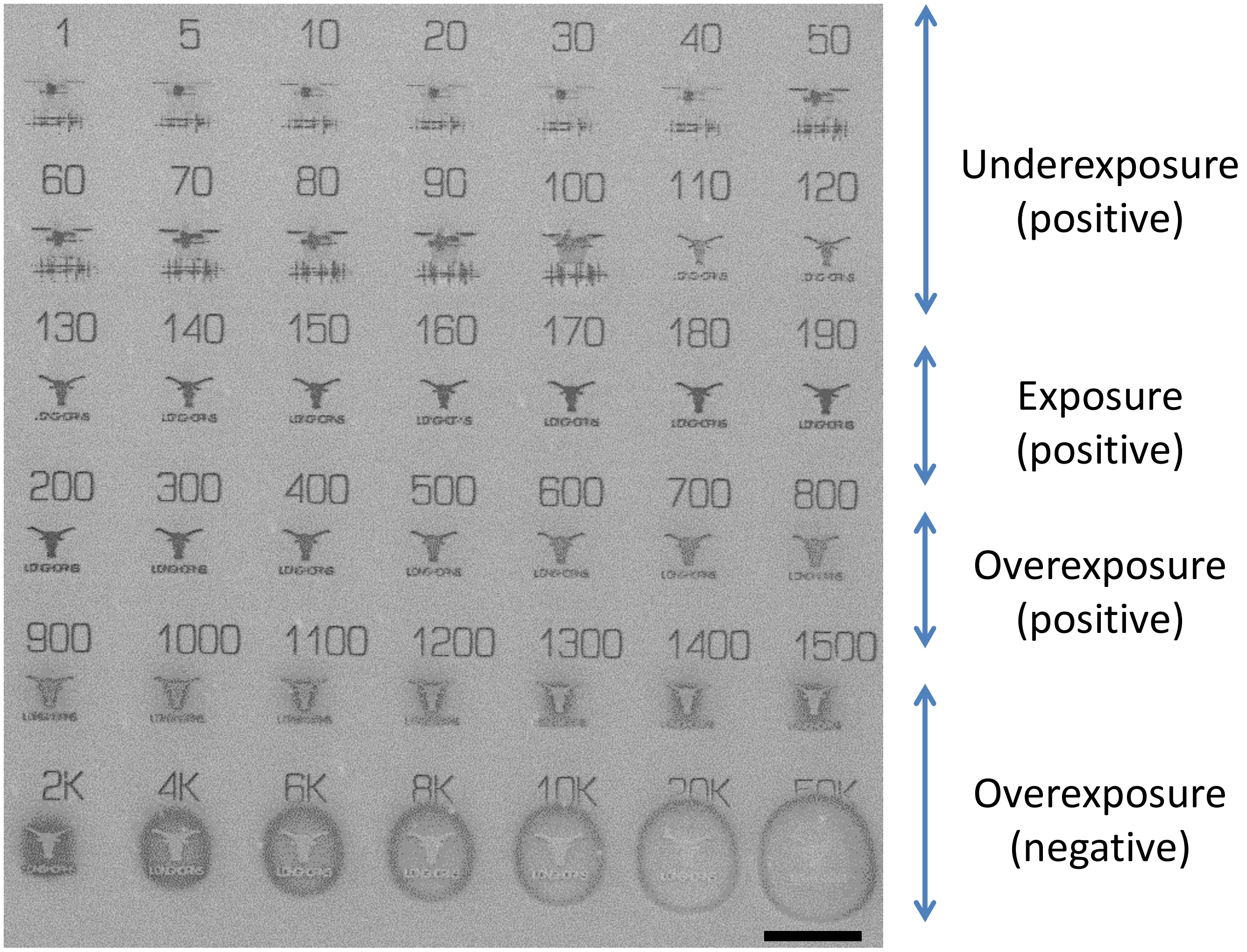

Supplementary Figure 1. SEM image of an array of “long horns” in increasing dosages, showing the different stages of electron-structure interactions. Scale bar, 5 µm.

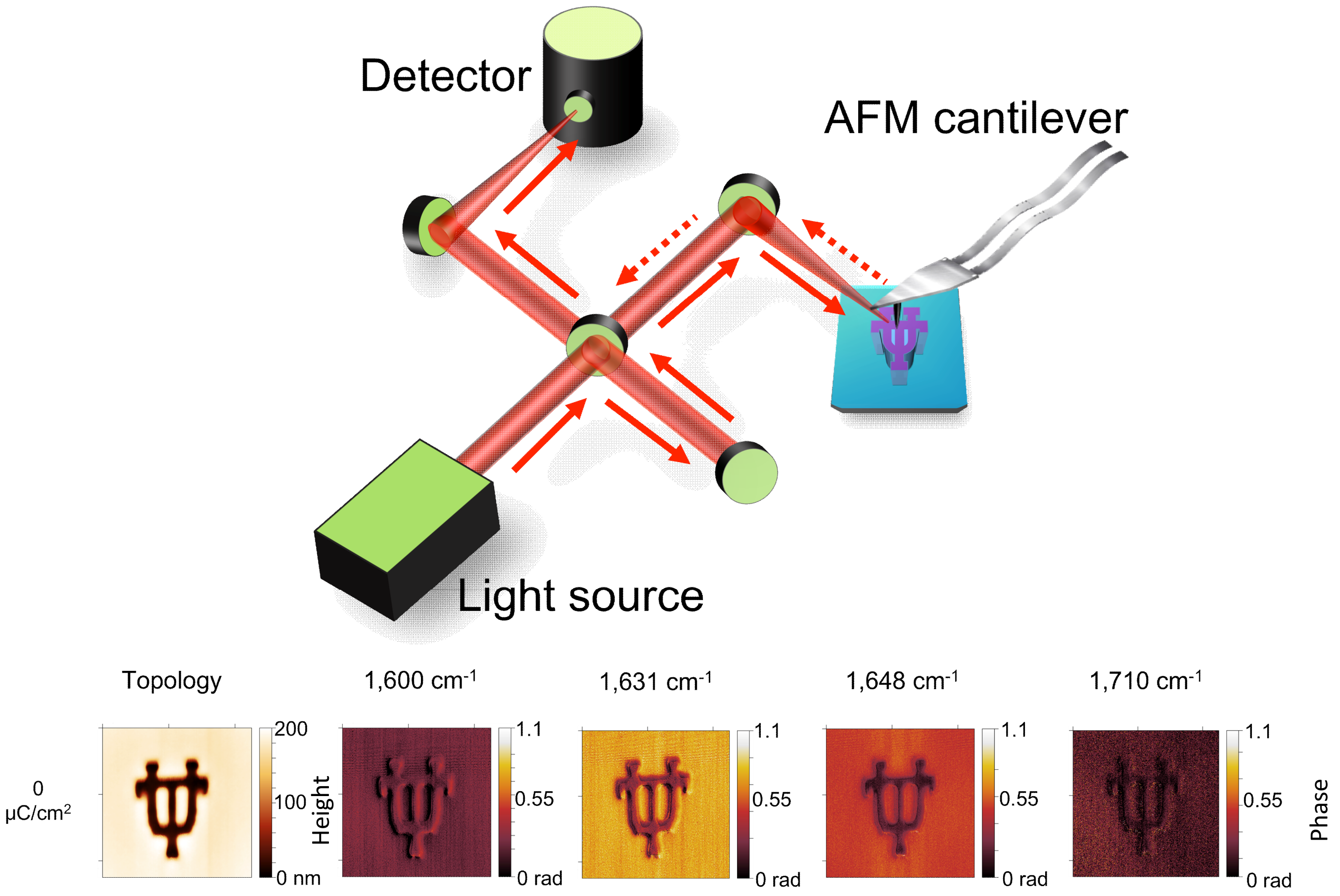

Supplementary Figure 2. IR nano-imaging: s-SNOM set-up employing a tunable single line IR quantum cascade laser (1,450 to 1,750 cm^{-1}) for tip illumination. The light backscattered from the tip is collected and analyzed with a Michelson interferometer operating in pseudo-heterodyne mode. Both amplitude and phase information is collected. The near-field interaction leads to a phase spectrum that resembles a familiar molecular absorbance band, while the near-field amplitude spectrum acquires a dispersive line shape similar to a far-field reflectivity spectrum.

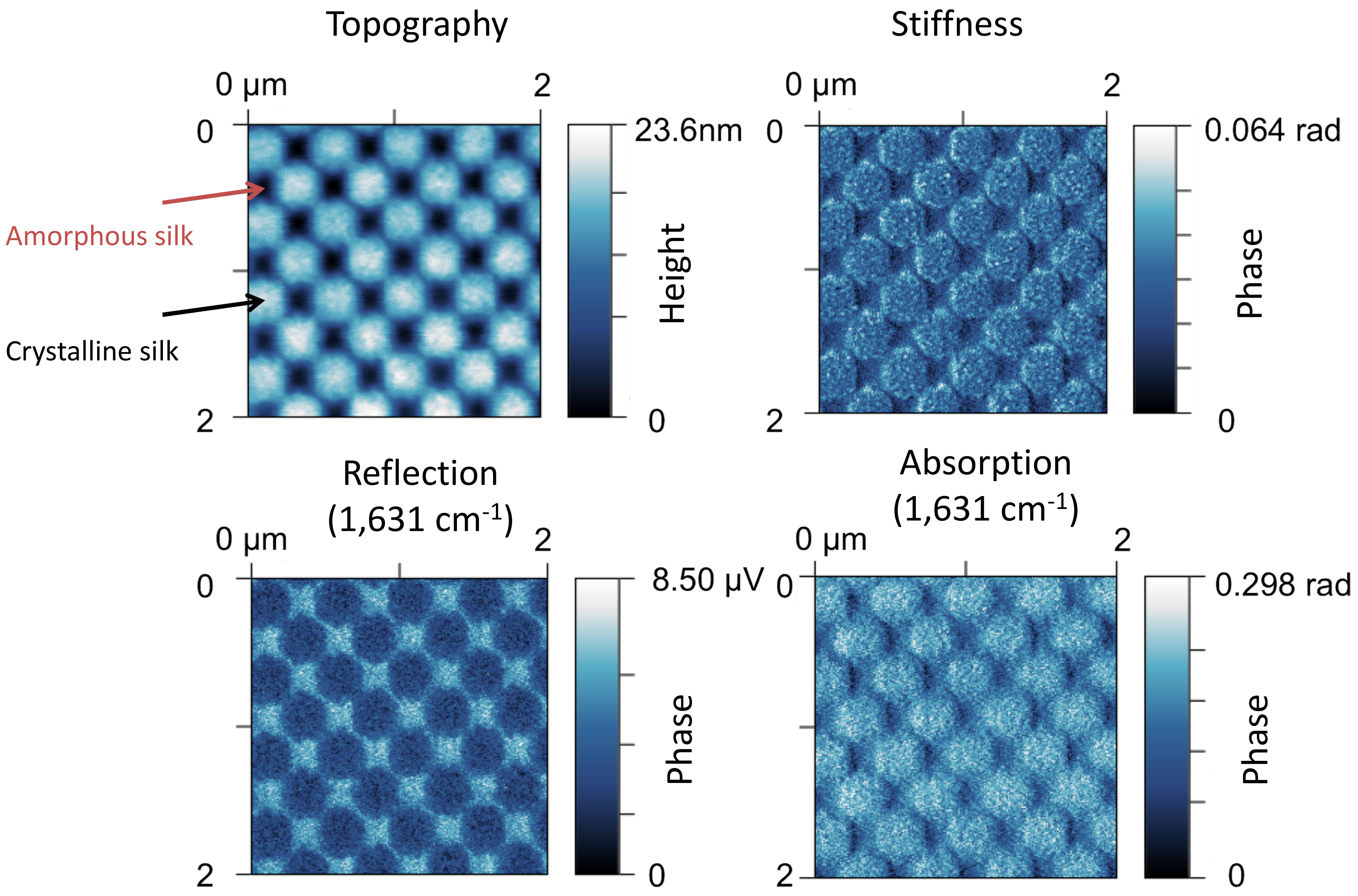

Supplementary Figure 3. Simultaneously monitor the topographical, mechanical and chemical properties of silk proteins (nanopatterned in check board, pitch: 250 nm) at the nanoscale using IR-SNOM.

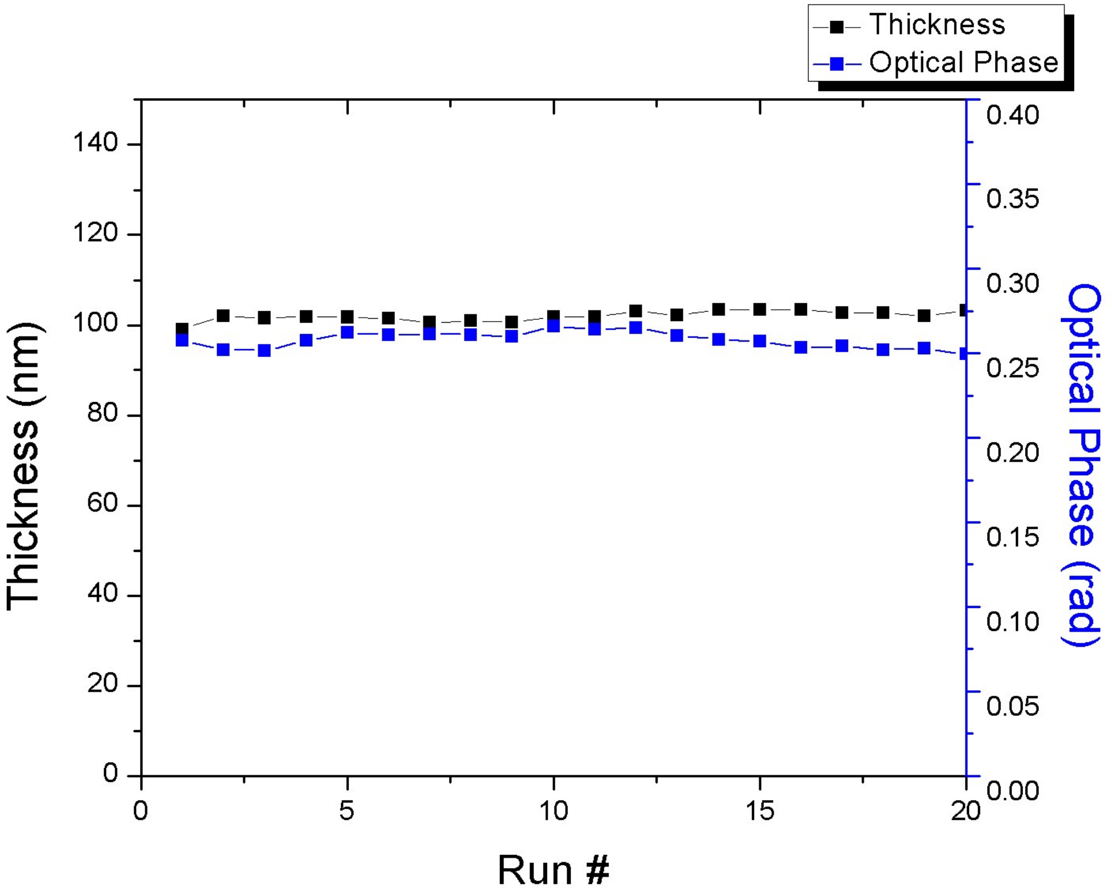

Supplementary Figure 4. A set of experiments to confirm the non-invasive nature of SNOM in our setup. Basically, after 20 runs/measurements, the sample remains intact (both thickness and IR signal, which corresponds to the structural conformation, for example, 1631 cm^{-1} for crosslinked beta-sheets in silk). Note that there are some minor variations which are due to (an AFM-based) system drift and are common. Experiment conditions: # of runs: 20; Laser power: ~ 4 mW; AFM tip free vibration amplitude: 67; Tapping amplitude: 53 nm; Wavenumber: 1631 cm^{-1}; Scan area: 3 µm by 1.5 µm; Scan speed: 4.5 µm/s; Scan lines: 100 lines.

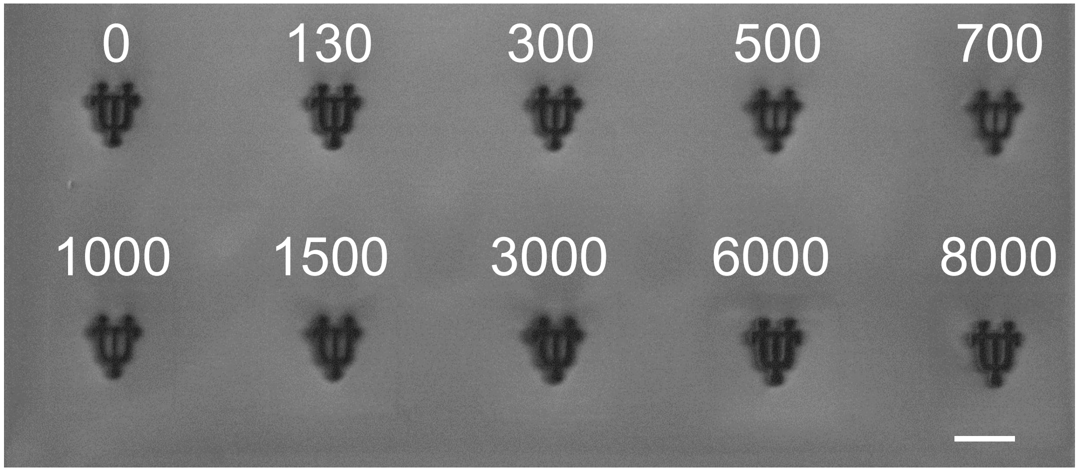

Supplementary Figure 5. SEM images of silk nanopatterns fabricated using EBL at the dosages ranging from 0 to 8,000 μC/cm^2. Scale bar, 2 μm.

Spectroscopic imaging

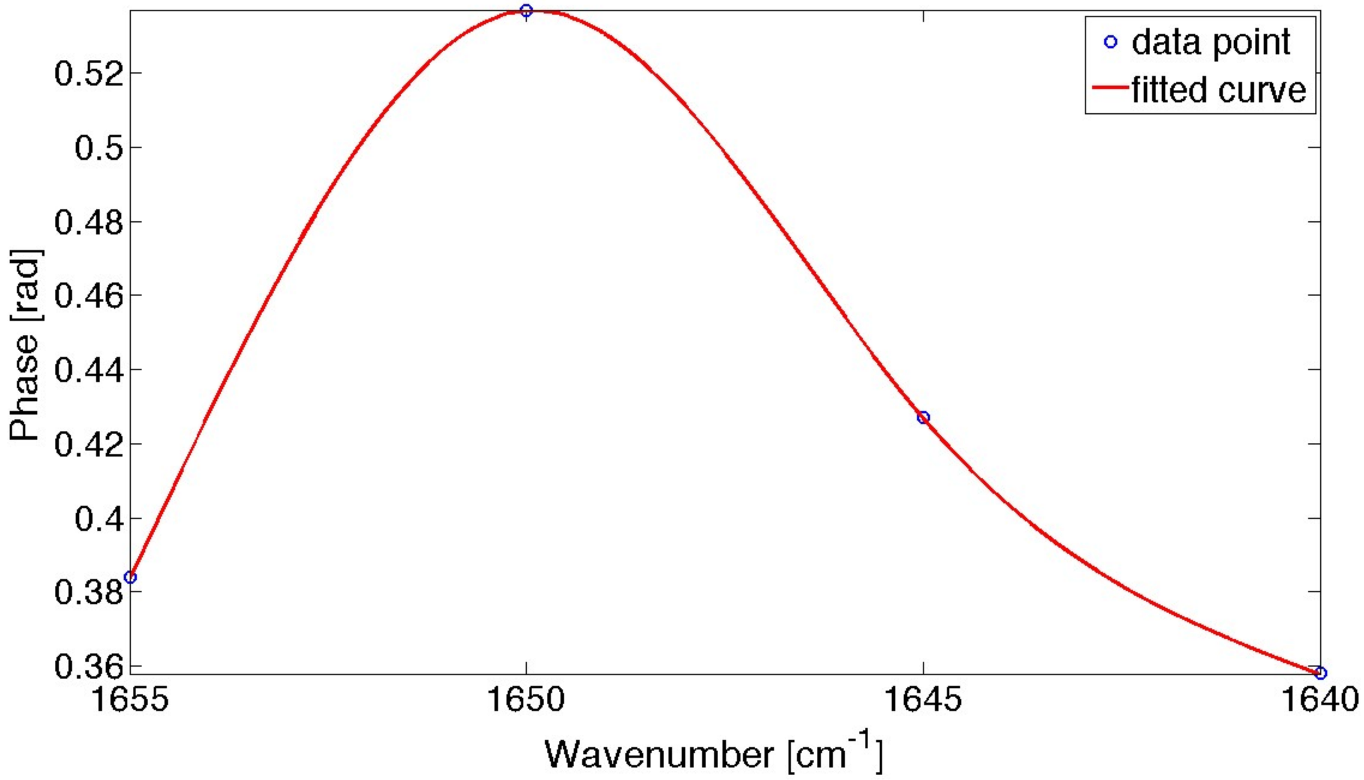

Supplementary Figure 6. Local infrared absorption spectra (symbols) depicting the normalized phase signal of the near-field signal of amorphous silk (i.e., rich in random coils) by sweeping the output wavenumber of the QCL and using nanospectroscopic imaging.

IR Reflectivity: 1,580 cm⁻¹

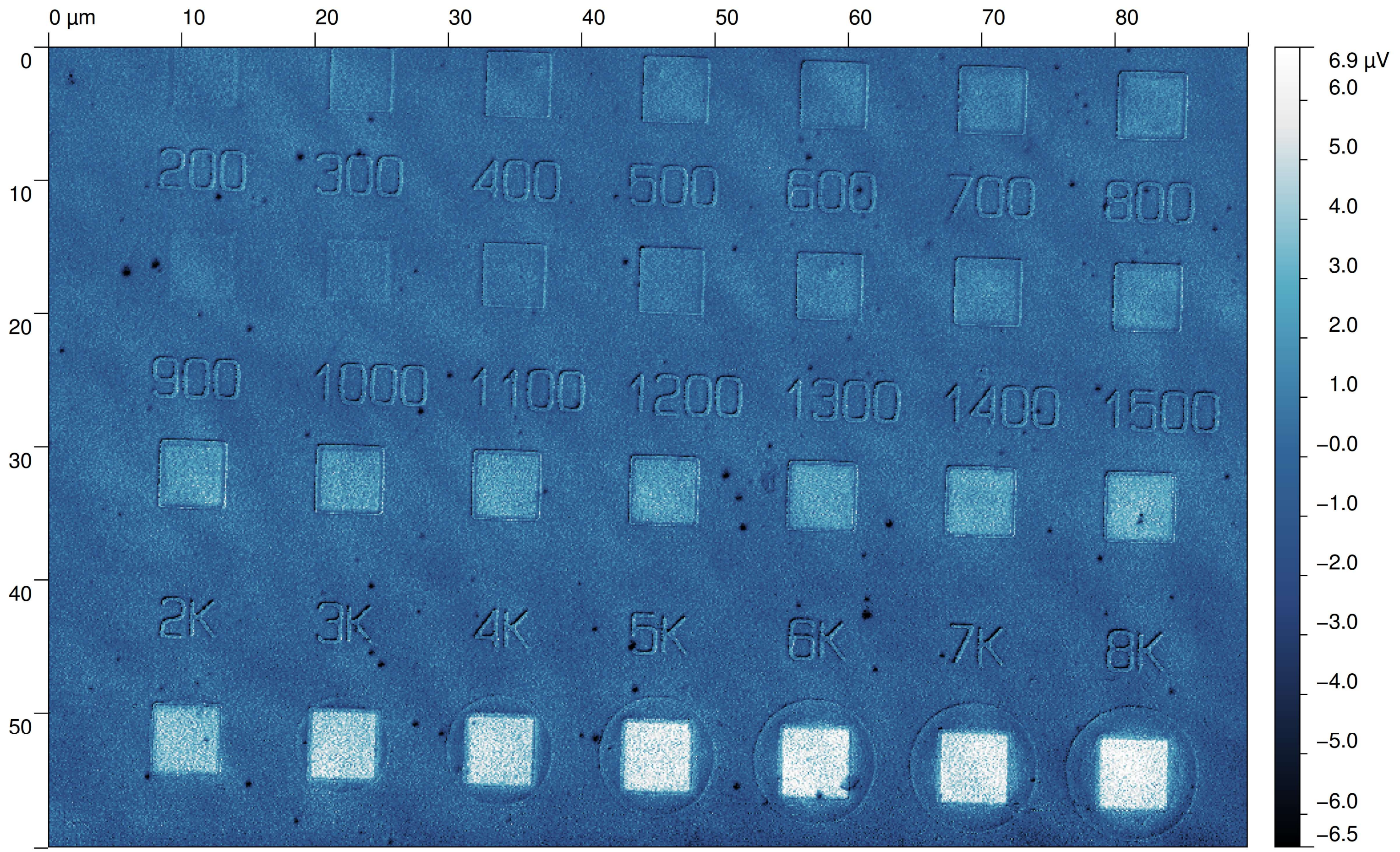

Supplementary Figure 7. IR reflection of crystalline silk proteins exposed at different dosages of electron radiation at 1,580 cm^{-1}, i.e., off-resonance of silk within the amide I vibration bands, which correlates with the electrical conductivities.

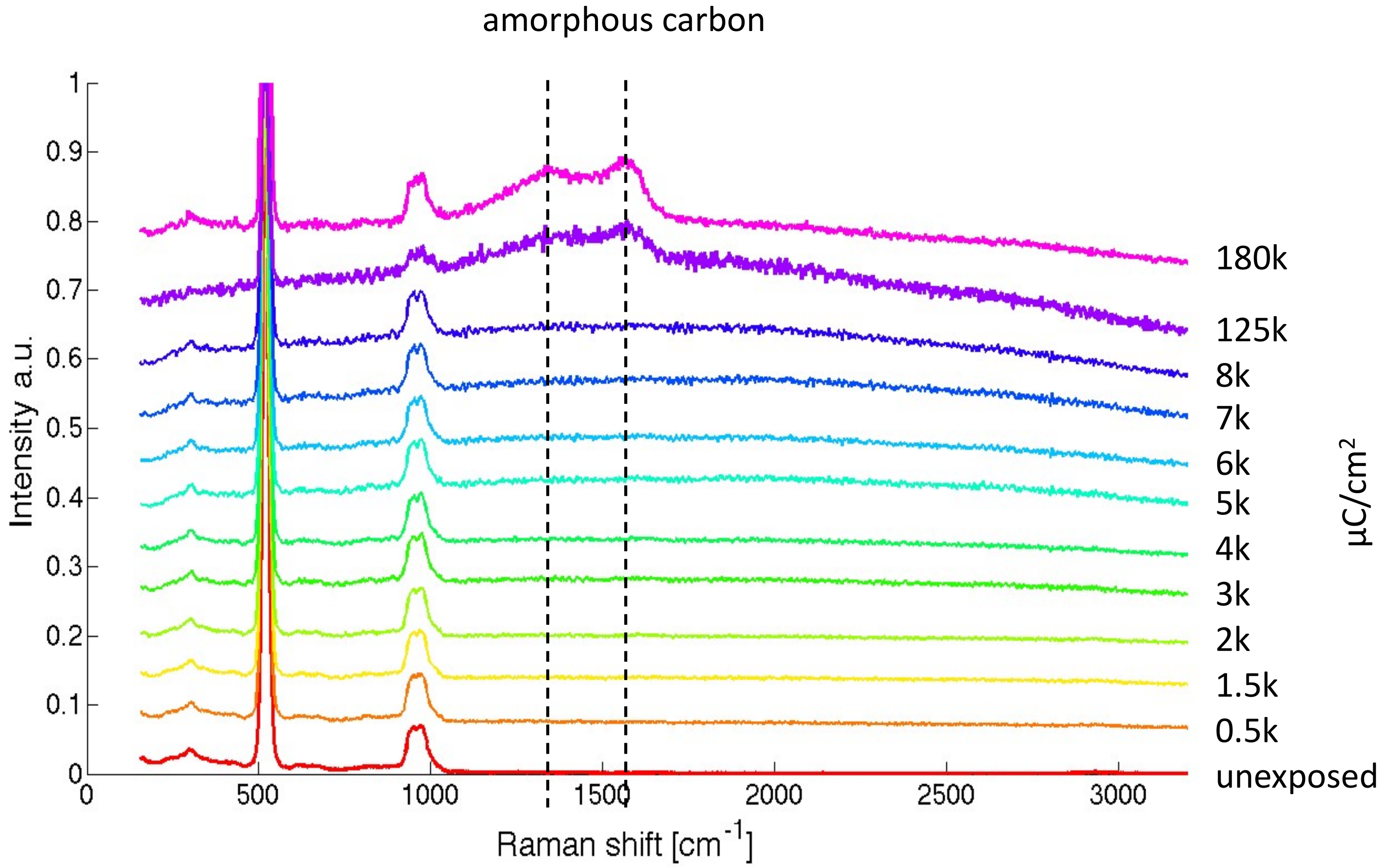

Supplementary Figure 8. Raman spectra of crystalline silk proteins exposed at different dosages of electron radiation.

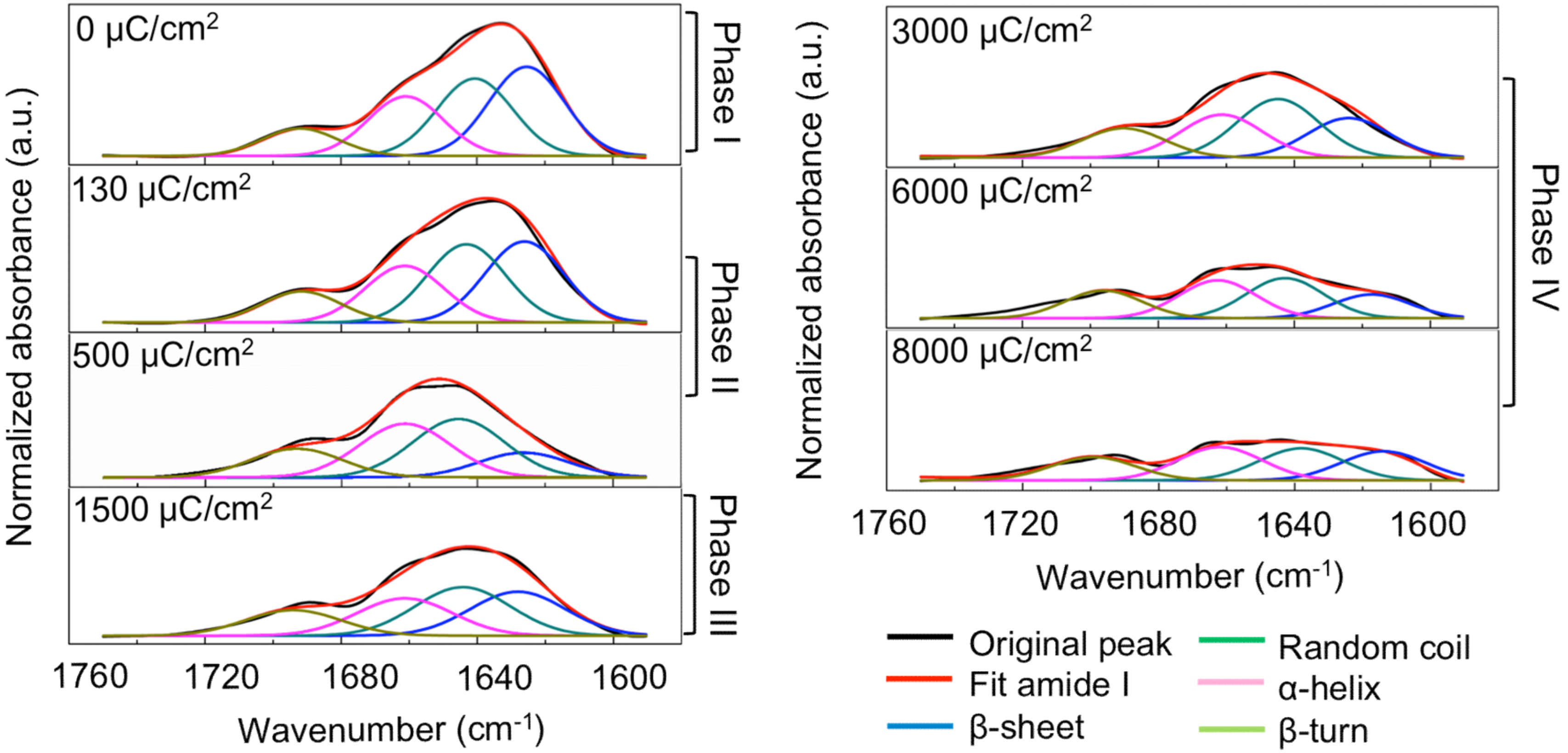

Supplementary Figure 9. Quantitation of the nanoIR spectra at different transition phases (in Figure 2b) using deconvolution.

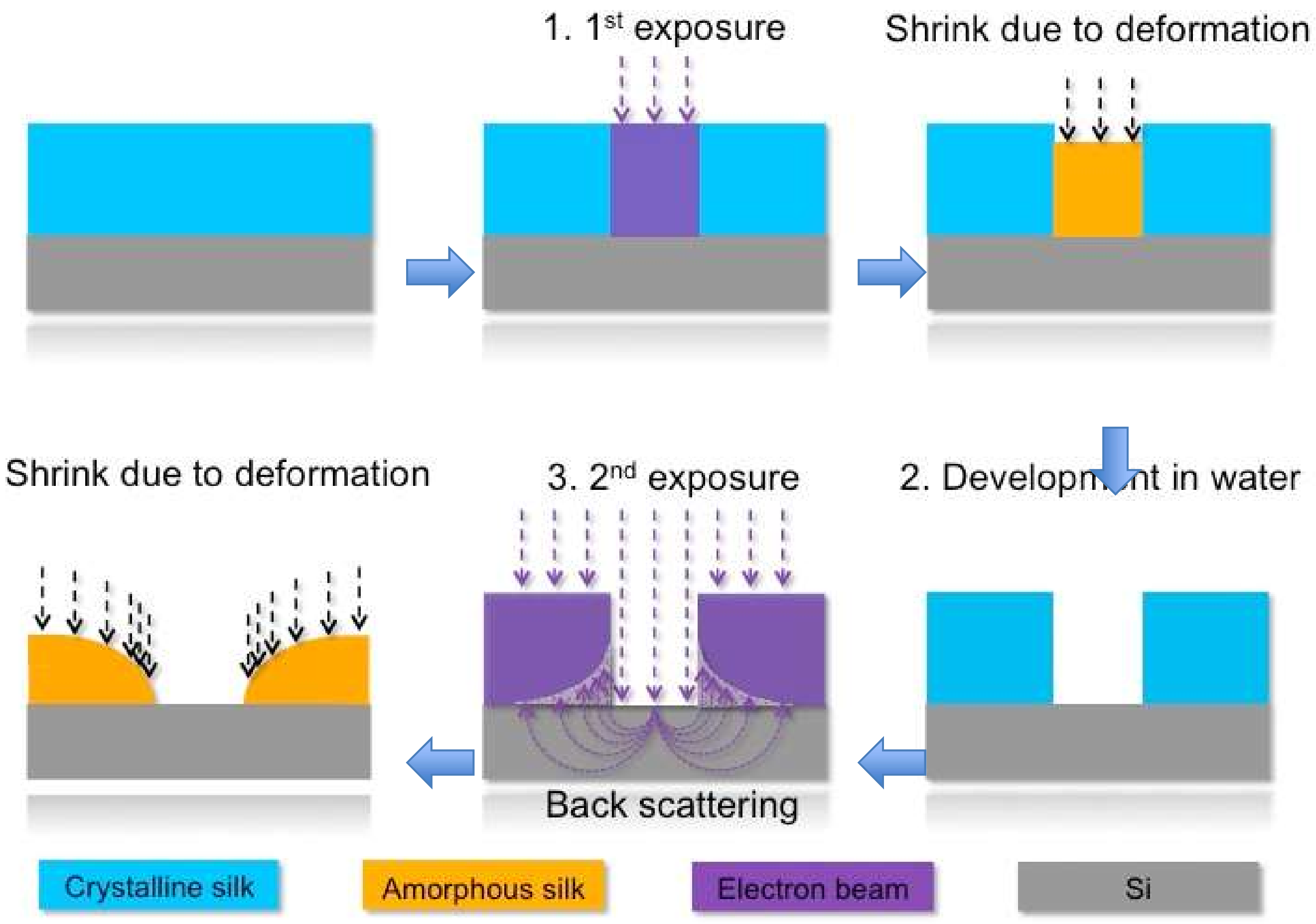

Supplementary Figure 10. Illustration of the electron-protein interaction over the interface of silk and silicon.

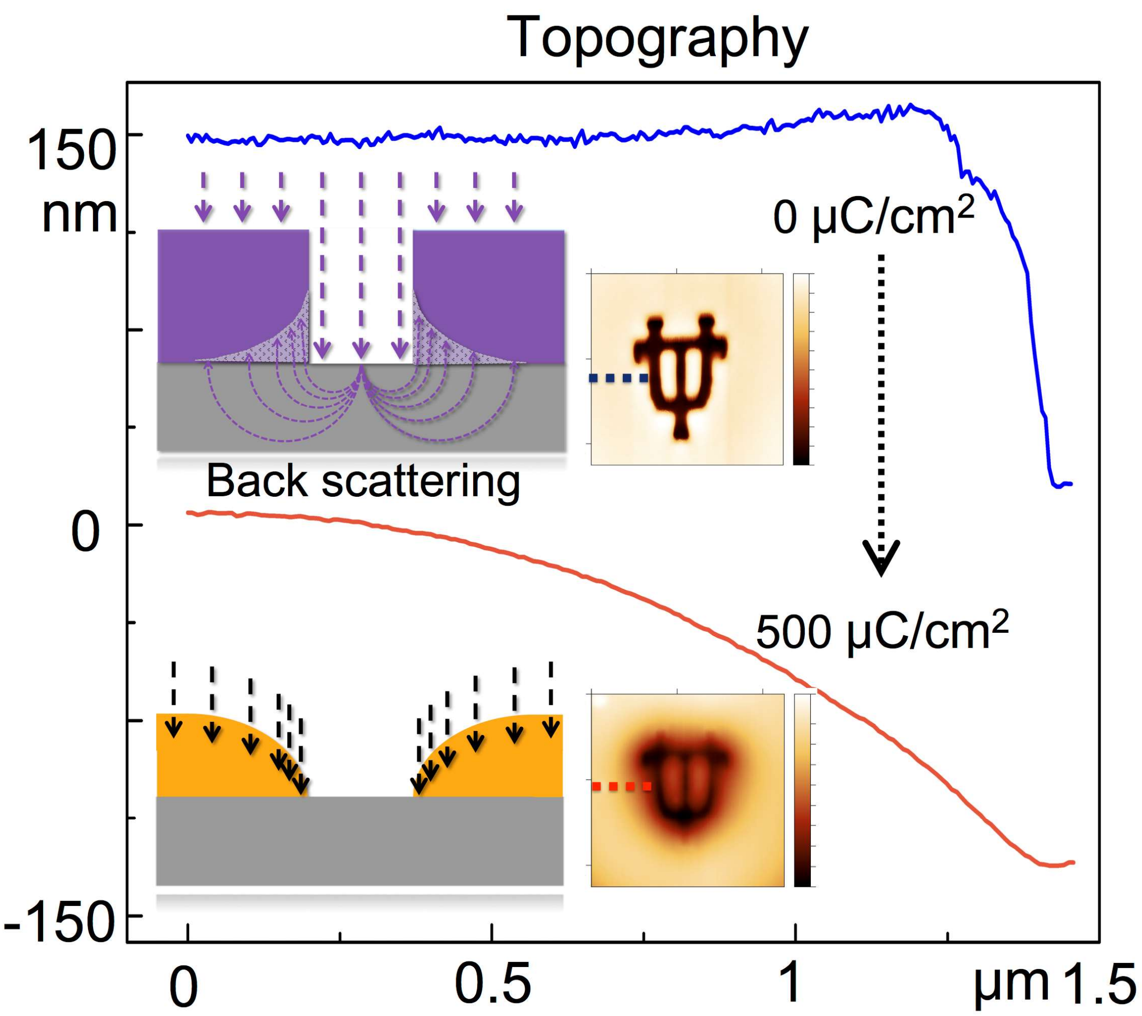

Supplementary Figure 11. Topographic diagrams illustrate the electron-protein interaction over the interface of silk and silicon characterized using s-SNOM. (Insets) The silk thin film undergoes a second exposure due to backscattered electrons from the adjacent silicon substrate and shrinks accordingly due to deformation, which becomes more prominent as gets closer towards silicon.

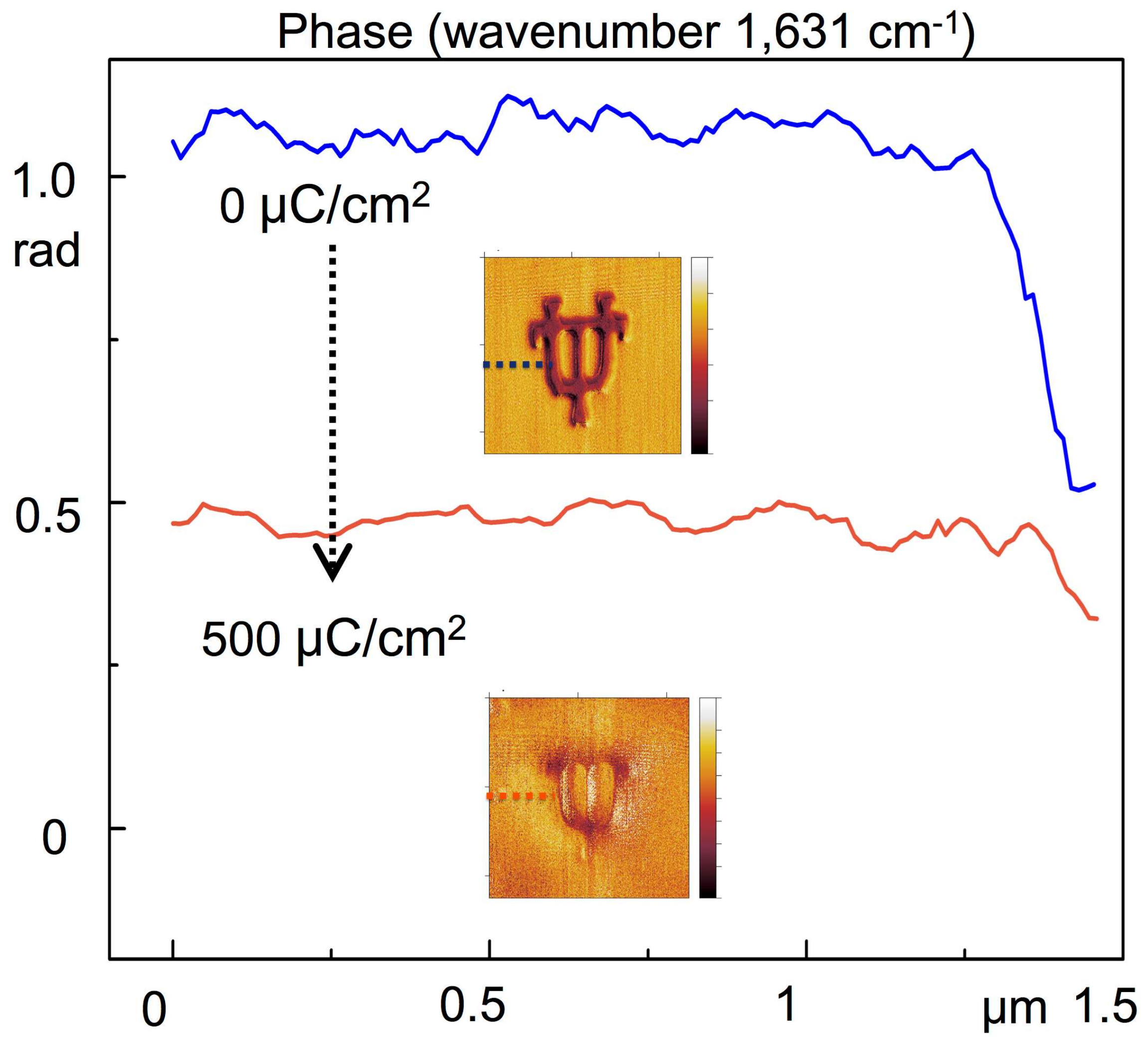

Supplementary Figure 12. Infrared near-field phase images confirm the complete deformation of β-sheets at the dosage of 500 μC/cm^2.

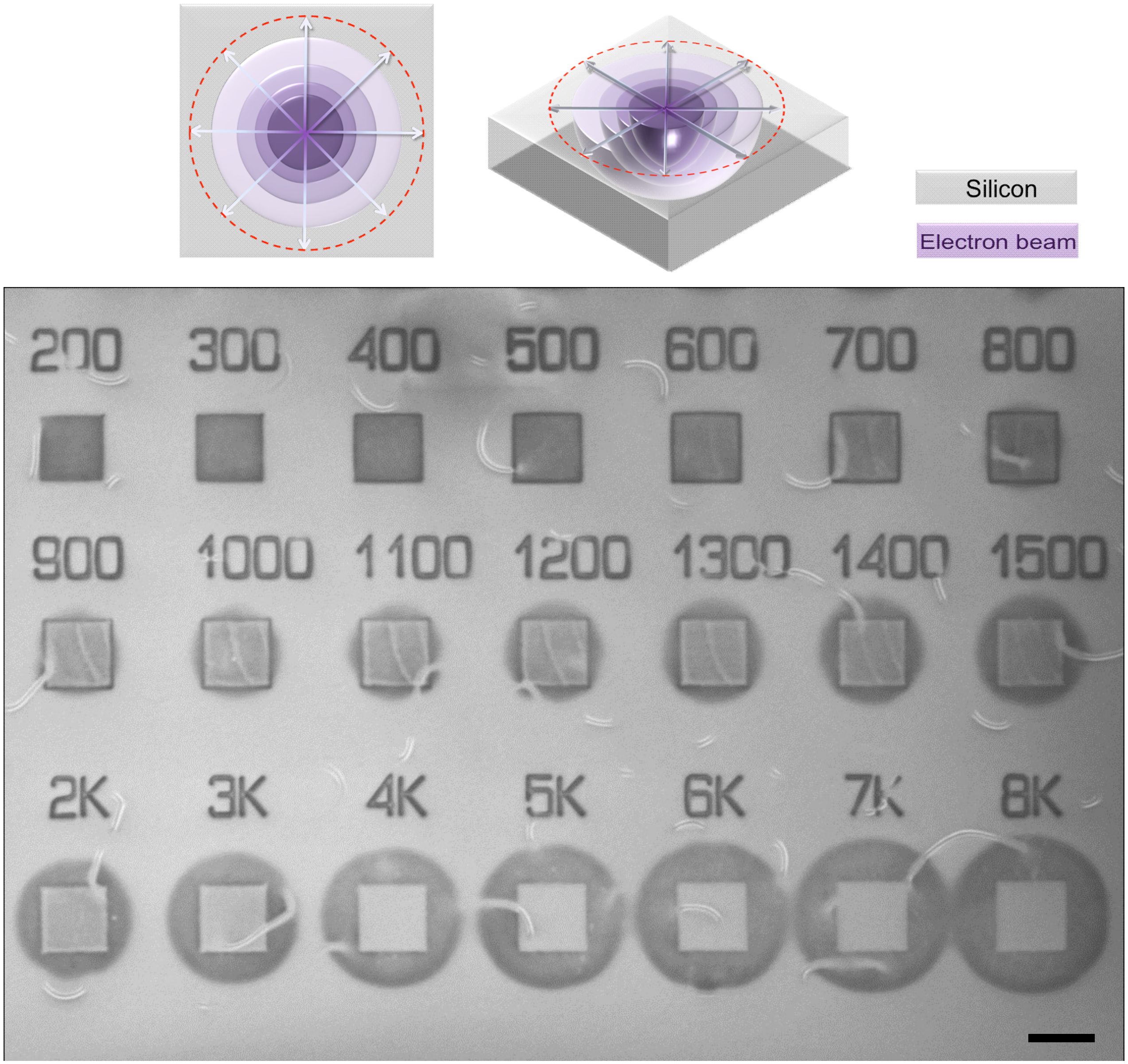

Supplementary Figure 13. SEM image of an array of EBL-fabricated squares (5µm * 5µm) in increasing dosages, showing the proximity effect due to backscattered electrons in silicon. Scale bar, 5 µm.

$$\beta_{\mu m} = 0.046 \frac{(V_{kV})^{1.75}}{\rho_{g/cm^3}} \qquad V_{kV} = 25,\ \rho_{g/cm^3} = 2.33$$

$$\beta_{\mu m} = 5.518$$

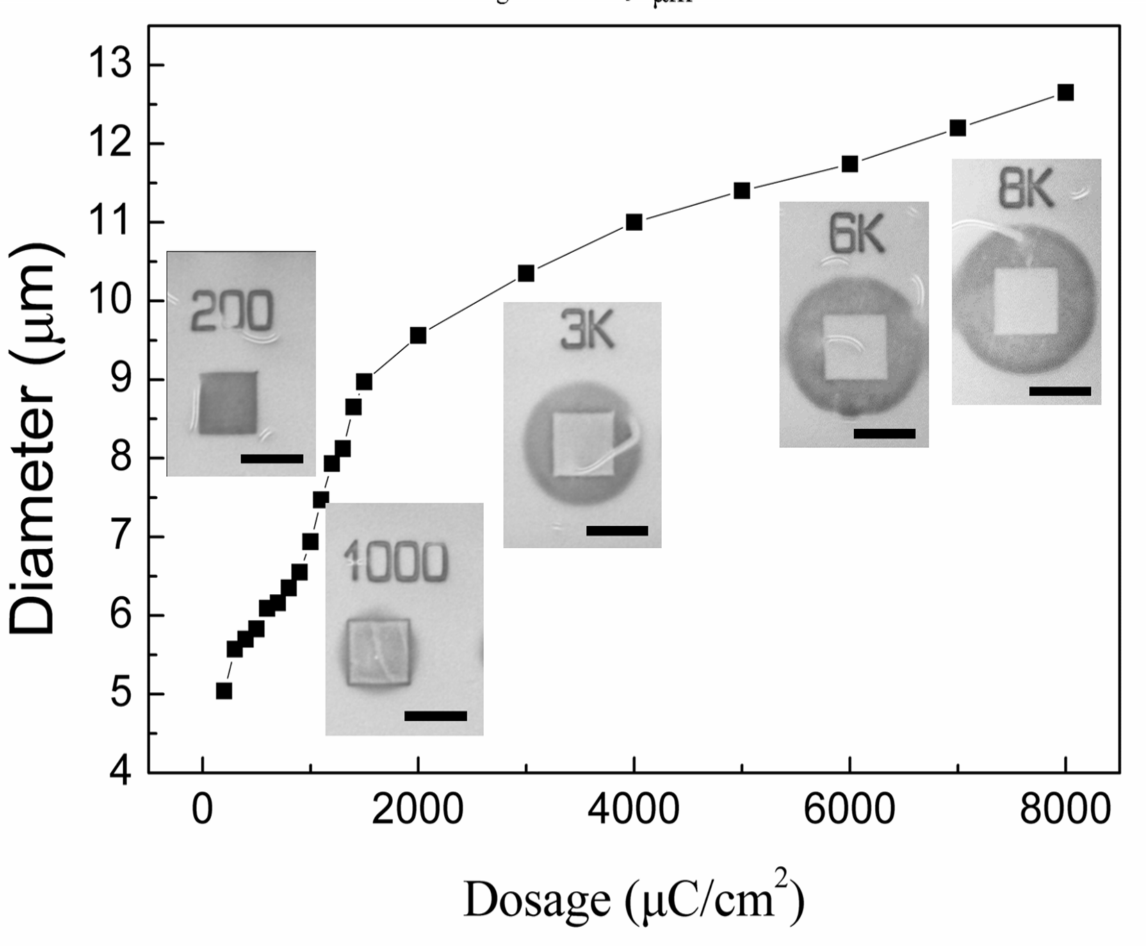

Supplementary Figure 14. Direct observation of stray exposure in silk proteins on silicon due to proximity effect, which is in reasonable agreement with theoretic calculations. Scale bar, 5 µm.

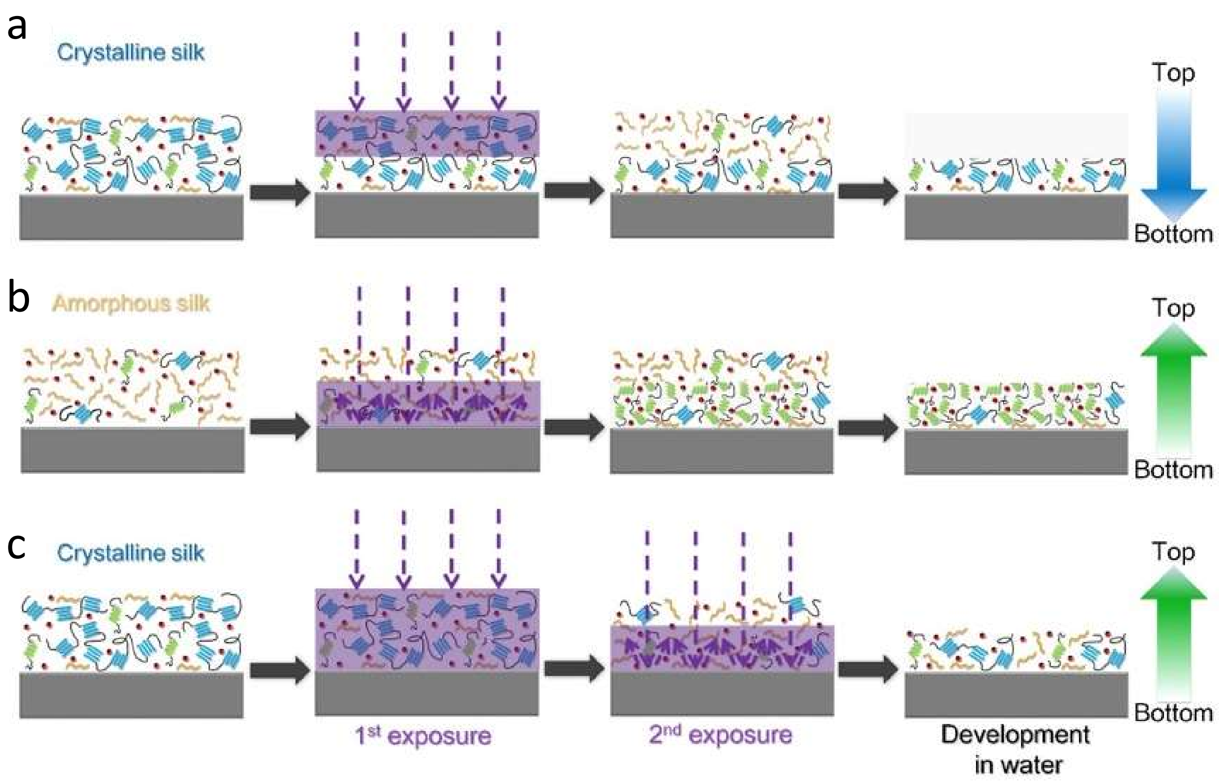

Supplementary Figure 15. (a) – (c) Three sets of silk nanostructures have been made in positive (row 1, nanosculpturing) and negative (row 2 and row 3, nanosintering) tones using EBL at various dosages. Schematic illustrations of different kinetics of electron-structure interaction in amorphous silk and crystalline silk.

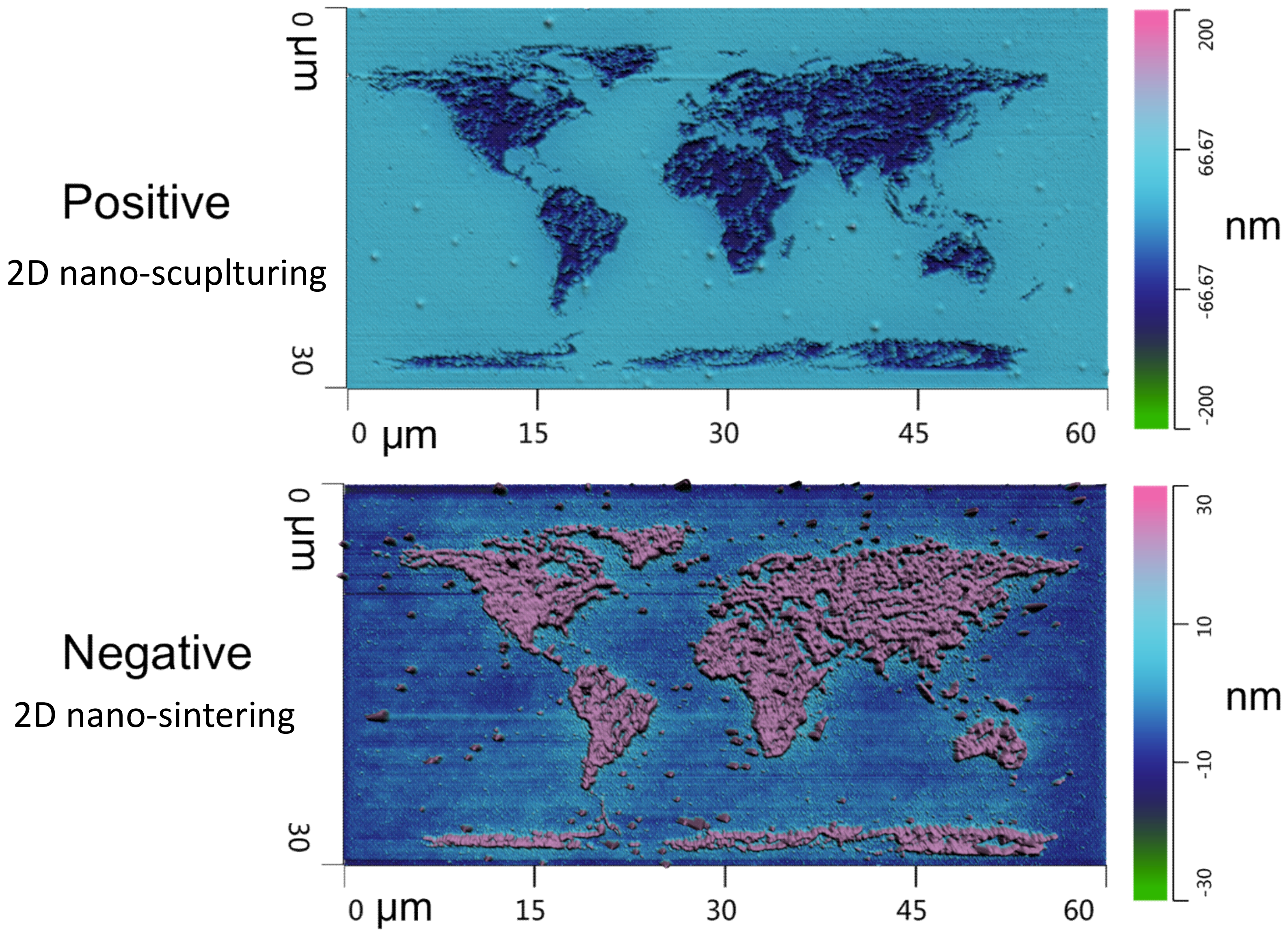

Supplementary Figure 16. 2D nanopatterning on silk using either positive (crystalline silk) or negative (amorphous silk) EBL.

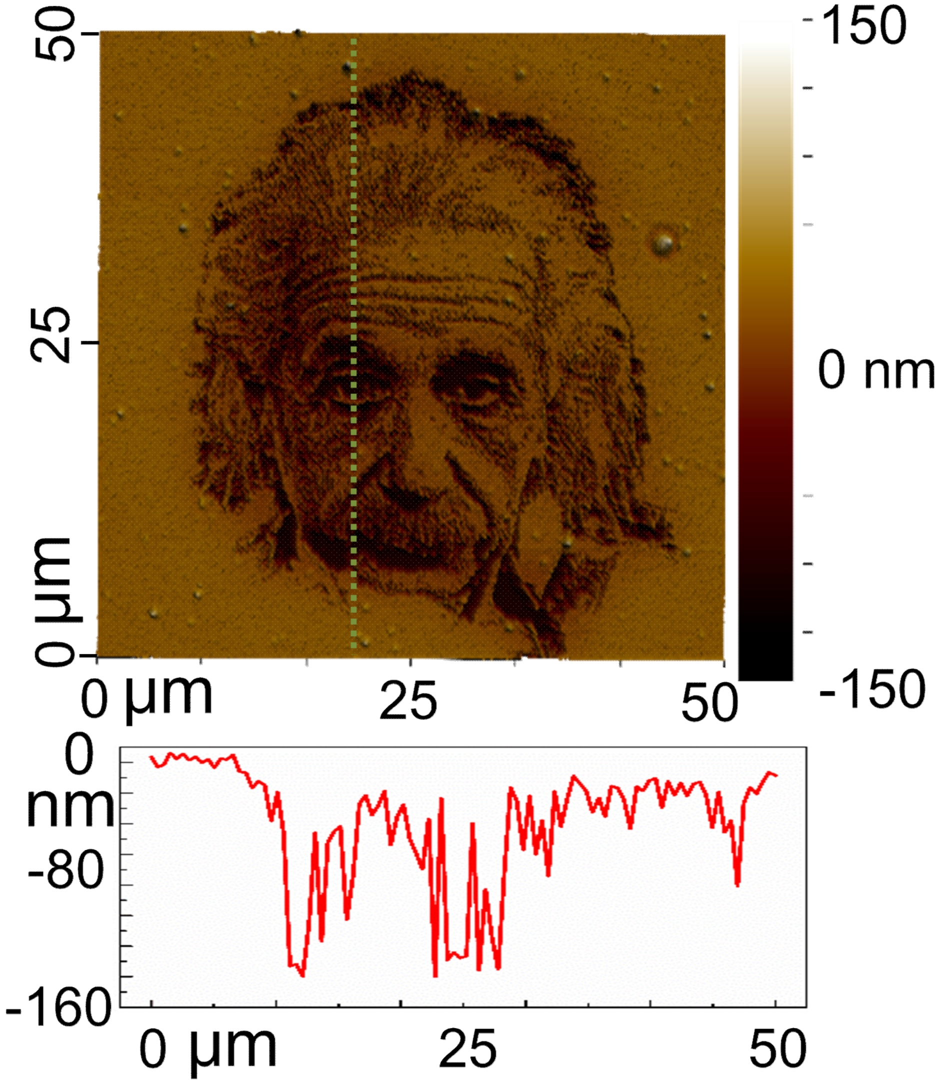

Supplementary Figure 17. AFM image of 3D nanosculpturing on crystalline silk fibroin proteins (as shown in **Figure 5m**) using a 16bit greyscale EBL in unprecedented detail.

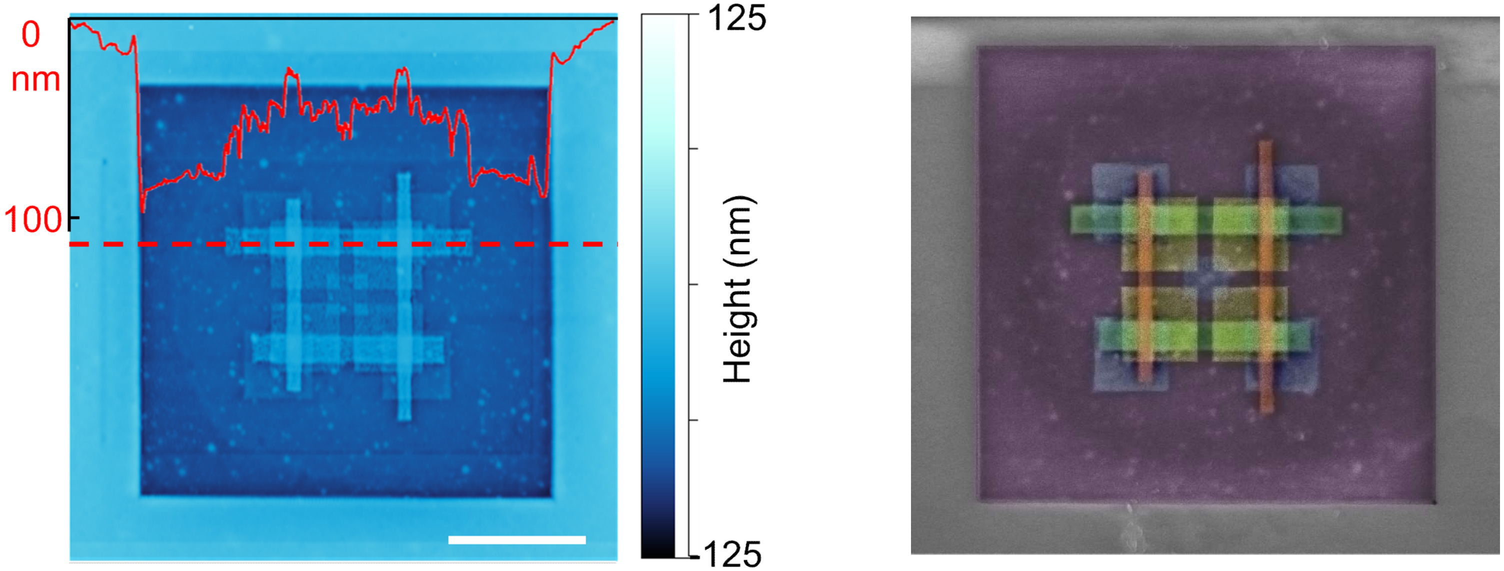

Supplementary Figure 18. AFM and SEM (false coloring) images of 3D nanosintering (in **Figure 5c**) using LbL EBL. Scale bar, 5 µm.

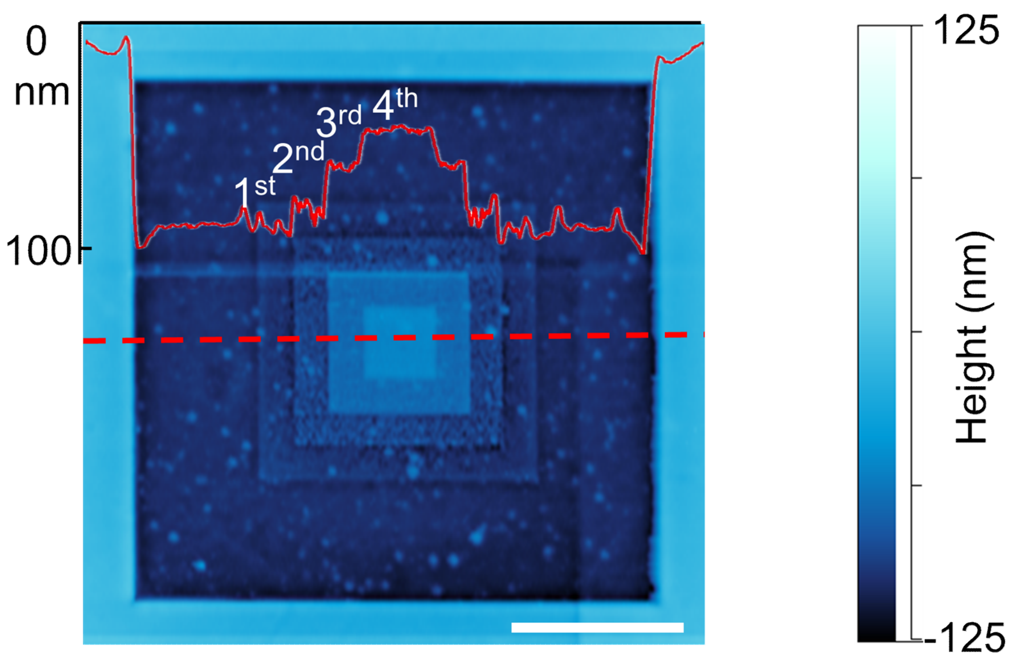

Supplementary Figure 19. AFM image of 3D nano-sintering of silk proteins using LbL EBL. Scale bar, 5 µm.
